# Coronary Ectasia and ST Elevation Myocardial Infarction Patients: Does Atherosclerosis Influence Periprocedural Management and Long‐Term Prognosis?

**DOI:** 10.1002/ccd.70142

**Published:** 2025-09-03

**Authors:** Victorine Fraichot, Jeanne Varlot, Florian Eggenspieler, Nassima Djaballah, Pierre Adrien Metzdorf, Edoardo Camenzind, Batric Popovic

**Affiliations:** ^1^ Department of Cardiology Université de Lorraine, CHRU‐Nancy Nancy France

**Keywords:** atherosclerotic coronary artery disease, coronary artery ectasia, myocardial infarction

## Abstract

**Background:**

Coronary artery ectasia (CAE) influences procedural outcomes in the context of ST‐elevation myocardial infarction (STEMI); however, its relationship with atherosclerotic coronary artery disease (ACAD) remains unclear.

**Aims:**

This study aimed to compare clinical and procedural characteristics, as well as outcomes, in patients with STEMI and CAE, with or without coexisting ACAD.

**Methods:**

Overall, 148 patients with STEMI and ectatic infarct‐related artery who underwent primary percutaneous intervention were included from 2003 to 2021. These patients were stratified based on the presence of atherosclerotic disease into two groups: patients with STEMI and isolated CAE (*n* = 74) and those with CAE and coexisting ACAD (*n* = 74).

**Results:**

Compared with patients in the isolated CAE group, those in the CAE and coexisting ACAD groups were older (65 vs. 58.4 year, *p* = 0.002), with no significant differences in cardiovascular risk factors or initial clinical presentation. Coronary angioplasty was performed more frequently in the CAE with coexisting ACAD group (90.5% vs. 63.7%, *p* < 0.001), with a higher stenting rate (73.0% vs. 48.6%, *p* = 0.004) and a trend toward less frequent distal embolization (35.3% vs. 52.9%, *p* = 0.057). No significant differences were observed between the two groups in troponin or CPK levels, nor in left ventricular ejection fraction at hospital discharge (48 ± 10% vs. 49 ± 10%, *p* = 0.569). At the 3‐year follow‐up, the overall MACE‐free rate was 85.1%, with no significant difference between the groups (83.8% vs. 78.4%, *p* = 0.487).

**Conclusion:**

Patients with STEMI and ectatic related infarct‐related artery who underwent primary PCI demonstrated distinct periprocedural characteristics depending on the presence or absence of coronary atherosclerosis. Further research is needed to determine optimal therapeutic management at discharge.

## 1 Introduction

Prompt revascularization to achieve optimal thrombolysis in myocardial infarction (TIMI) flow grade remains the key objective of STEMI management, significantly influencing patient outcomes [1]. While a TIMI 3 flow grade is achieved in most procedures [2, 3], abnormal vessel dilatation associated with a disturbed coronary flow, such as coronary ectasia, may influence optimal results [4]. The pathogenesis of coronary artery ectasia (CAE) has not been fully elucidated and may be classified into different types [5, 6]: (1) congenital, (2) atherosclerotic (acquired, in the setting of atherosclerotic coronary artery disease [ACAD]), or (3) isolated acquired related to inflammation or connective tissue disorders) [7, 8]. Although atherosclerosis seems to be the underlying mechanism of coronary dilation in many cases, few studies have compared the results of revascularization according to CAE pathogenesis. This study aimed to investigate the prevalence, periprocedural impact, and long‐term prognosis of coexisting coronary atherosclerosis in patients with STEMI and ectatic infarct‐related arteries (IRA).

## 2 Methods

### 2.1 Patient Selection

This retrospective observational cohort study included all patients with STEMI and ectatic IRAs who underwent primary percutaneous coronary intervention (PCI) at our center between 2003 and 2021. STEMI was based on the following criteria*:* (1) continuous chest pain for at least 20 min; (2) ST‐segment elevation ≥ 1 mm (0.1 mV) in ≥ 2 contiguous leads on the 12‐lead electrocardiogram (ECG); or (3) left bundle‐branch block treated within 24 h of pain onset.

### 2.2 Angiographic Analysis and Definitions

CAE was defined as the dilatation of a coronary segment with a diameter 1.5 times higher than that of the normal adjacent segments [8]. Additionally, we specified ectasia as either diffuse involving 50% or more of the length of the affected artery or segmental dilation. Moreover, patients were categorized as having isolated CAE or mixed lesions according to the following criteria: isolated CAE when there was no evidence for atherosclerosis and CAE with coexisting ACAD when, in addition to the dilated segments, there was classical evidence for atherosclerotic changes (plaque formation or minor atheroma ≥ 25% of the lumen narrowing). To minimize the subjectivity inherent to visual angiographic assessment, we systematically performed quantitative coronary angiography (QCA), which enabled an objective measurement of vessel dimensions. During the primary PCI procedure, coronary angiography was repeated in the working projection, and at least two orthogonal angiographic views were used to evaluate the TIMI flow grade at the end of the procedure. Distal embolization was identified by an abrupt “cut‐off” in one of the coronary branches of the infarct related artery distal to the angioplasty site [9].

### 2.3 ECG Analysis

ECG were recorded upon arrival at the catheterization laboratory (pre‐procedural ECG) and in the coronary care unit 60–90 min after primary PCI/angiography (postprocedural ECG). Pre‐procedural ST‐segment deviation and postprocedural residual ST‐segment deviation were used to assess the ST‐segment deviation. The degree of ST‐segment resolution (STR) was calculated by dividing the extent of ST‐segment deviation on the post‐procedural ECG by that on the pre‐procedural ECG, and the result was expressed as a percentage [10].

### 2.4 Treatment Modalities

Primary PCI was performed using standard techniques, with either the radial or the femoral approach selected based on the physician's preference.

Before the procedure, all patients received 250–500 mg of intravenous acetylsalicylic acid. Additionally, a P2Y12 inhibitor was administered as early as possible during the prehospital phase which was continued as a daily regimen for the first year. A GP2b/3a inhibitor (GPI) was added to the catheterization laboratory at the discretion of the physician.

### 2.5 Outcomes

Clinical outcomes included major adverse cardiovascular events (MACE) such as non‐fatal myocardial infarction, stent thrombosis and all‐cause mortality at the 3‐year follow‐up, which was performed via direct telephonic contact with the patient's general physician to complete comprehensive standardized questionnaires. In the case of death, the patient's physician or hospital records were consulted to determine the cause.

The study protocol and informed consent forms were approved by the Institutional Ethics Committee of the University Hospital of Nancy (France).

### 2.6 Statistical Analysis

Patients' baseline characteristics and outcomes were described using frequencies and percentages for categorical variables and mean and standard deviations (SDs) for continuous variables. Comparisons between groups were performed using the *χ*² test or Fisher's exact test for categorical variables, depending on the conditions of application, and Student's *t*‐test for continuous variables. To compare event‐free survival between groups, we employed the Tarone−Ware test [11]. The statistical significance level was set at 0.05. All analyses were conducted using the R software Version 4.3.2 (R Foundation for Statistical Computing, Vienna, Austria).

## 3 Results

The baseline patient characteristics are presented in Table 1. The study included 148 patients. Patients with CAE and coexisting ACAD‐isolated CAE were older (mean age: 65 vs. 58.4 years, *p* = 0.002) than those with isolated CAE; however, no difference in cardiovascular risk factors or medical history was observed. Angiographic and procedural characteristics are detailed in Table 2.

**Table 1:** Baseline characteristics of patients according to presence of a atherosclerotic coronary artery disease.

	All patients	CAE with ACAD	Isolated CAE	*p* value
	*n* = 148	*n* = 74	*n* = 74	
*Socio‐demographic characteristics*				
Age (years)	61.7 ± 13.1	65 ± 12.3	58.4 ± 13.2	0.002
Male sex (%)	122 (82.4)	64 (86.5)	58 (78.4)	0.28
*Cardiovascular factors*				
Smoking (%)	70 (47.3)	31 (41.9)	39 (52.7)	0.25
Hypertension (%)	76 (51.4)	41 (55.4)	35 (47.3)	0.41
Diabetes mellitus (%)	20 (13.5)	13 (17.6)	7 (9.5)	0.23
Dyslipidemia (%)	62 (41.9)	34 (45.9)	28 (37.8)	0.40
*Medical history*				
Chronic renal disease (%)	3 (2.0)	1 (1.4)	2 (2.7)	1.00
Atrial fibrillation (%)	8 (5.4)	5 (6.8)	3 (4.1)	0.72
Previous acute coronary syndrome (%)	11 (7.4)	7 (9.5)	4 (5.4)	0.53
Previous coronary angioplasty (%)	8 (5.4)	5 (6.8)	3 (4.1)	0.72
*At hospital admission*				
Delay 1st symptoms‐balloon (hours)	4.7 ± 3.3	4.4 ± 3.3	4.9 ± 3.2	0.39
Cardiogenic shock (%)	14 (9.5)	8 (10.8)	6 (8.1)	0.78
Cardiac arrest (%)	17 (11.5)	8 (10.8)	9 (12.2)	0.99
Acute heart failure without cardiogenic shock (%)	16 (10.8)	10 (13.5)	6 (8.1)	0.43

Abbreviation: ACAD, atherosclerotic coronary artery disease.

**Table 2:** Angiographic and procedural characteristics according to presence of a atherosclerotic coronary artery disease.

	All patients	CAE with ACAD	Isolated CAE	*p* value
	*n* = 148	*n* = 74	*n* = 74	
*Angiographic features*				
No. of vessels with ectasia	1.7 ± 0.8	1.6 ± 0.7	1.7 ± 0.8	0.54
*Culprit lesion location*				
Left main	8 (5.4)	4 (5.4)	4 (5.4)	1.0
Left anterior descending artery	43 (29.1)	25 (33.8)	18 (24.3)	0.28
Left circumflex	15 (10.1)	11 (14.9)	4 (5.4)	0.1
Right coronary artery	82 (55.4)	34 (45.9)	48 (64.9)	0.03
Stent thrombosis (%)	5 (3.4)	1 (1.4)	4 (5.4)	0.36
Pre‐procedural TIMI flow grade 3 (%)	27 (18.2)	14 (18.9)	13 (17.6)	0.99
Lesion diameter (mm)	4.2 ± 1.3	3.8 ± 0.7	4.6 ± 1.6	< 0.001
Lesion length (mm)	55.6 ± 31.8	53.6 ± 32.4	57.6 ± 31.3	0.44
*Type of ectasia*				
Diffuse ectasia (%)	93 (62.8)	42 (56.8)	51 (68.9)	0.17
Markis classification				
I	52 (35.1)	23 (31.1)	29 (39.2)	0.39
II	20 (13.5)	13 (17.6)	7 (9.5)	0.23
III	35 (23.6)	16 (21.6)	19 (25.7)	0.70
IV	41 (27.7)	22 (29.7)	19 (25.7)	0.71
*Procedural features*				
Coronary angioplasty (%)	114 (77.0)	67 (90.5)	47 (63.7)	< 0.001
Coronary angioplasty with stenting (%)	89 (61.0)	54 (73.0)	35 (48.6)	0.004
Direct stenting (%)	50 (55.6)	32 (57.1)	18 (48.6)	0.55
Number of stents	1.2 ± 0.4	1.1 ± 0.4	1.2 ± 0.5	0.29
Stent length (mm)	23.5 ± 13.2	22.5 ± 14.2	24.8 ± 12	0.43
Stent diameter (mm)	3.4 ± 0.7	3.3 ± 0.6	3.4 ± 0.8	0.61
Delayed stenting (%)	17 (11.5)	10 (13.5)	7 (9.5)	0.60
Thrombo‐aspiration (%)	49 (33.3)	18 (24.7)	31 (41.9)	0.04
Distal Embolization (%)	61 (44.2)	24 (35.3)	37 (52.9)	0.06
GpB3a inhibitors use	80 (54.1)	33 (44.6)	47 (63.5)	0.03
Postprocedural TIMI flow grade 3 (%)	104 (70.3)	54 (73.0)	50 (67.6)	0.59
*Electrocardiogram*				
ST segment resolution: (mean %)	59 ± 35	64 ± 35	54 ± 35	0.11
Complete ST segment resolution (*n*, %)	39 (26.4)	22 (29.7)	17 (23.0)	0.46

Abbreviations: ACAD, atherosclerotic coronary artery disease; GPI, glycoprotein inhibitors; STEMI, ST‐elevation myocardial infarction; STR, ST‐regression; TIMI, thrombolysis in myocardial infarction.

Compared to the isolated CAE group, patients with CAE and coexisting ACAD had a larger culprit lesion diameter (4.6 ± 1.6 mm vs. 3.8 ± 0.7 mm, *p* < 0.001), but no significant differences were observed in lesion length or lesion ectasia ratio. During the primary PCI procedure, coronary angioplasty was more frequently performed in the CAE with coexisting ACAD group (90.5% vs. 63.7%, *p* < 0.001) with a higher stenting rate (73.0% vs. 48.6%, *p* = 0.004). Although thromboaspiration (41.9% vs. 24.7%, *p* = 0.041) and GPI use (63.5% vs. 44.6%, *p* = 0.032) were more common in the isolated CAE group, there was a trend toward more frequent distal embolization in this group.

Myocardial perfusion, as evidenced by ECG analysis, showed complete STR resolution 1 h after the index PCI procedure in approximately one‐quarter of the patients in the entire population, with no significant difference between the two groups.

The clinical and biological assessments at initial hospitalization are shown in Table 3.

**Table 3:** In hospital course according to presence of a significant atherosclerotic coronary artery disease.

	All patients	CAE with ACAD	Isolated CAE	*p* value
	*n* = 148	*n* = 74	*n* = 74	
In hospital death	9 (6.1)	5 (6.7)	4 (5.4)	0.731
Right ventricular MI (%)	7 (4.7)	3 (4.1)	4 (5.4)	1.000
ECMO (%)	3 (2.0)	2 (2.7)	1 (1.4)	0.379
IABP (%)	8 (5.4)	5 (6.8)	3 (4.1)	0.716
BARC 2 bleeding (%)	14 (9.5)	8 (10.8)	6 (8.1)	0.779
Acute renal injury (%)	8 (5.4)	3 (4.1)	5 (6.8)	0.716
Biology:				
Troponin peak (µg/L)	54.4 ± 35.1	50.5 ± 36.5	58 ± 33.8	0.30
Troponin US peak (ng/L)	115 040 ± 167 114	130 914 ± 207 547	93 610 ± 88 466	0.45
CPK peak (UI/L)	2210 ± 1905	2081 ± 2020	2338 ± 1790	0.44
LVEF at discharge: (mean: %)	49 ± 10	48 ± 10	49 ± 10	0.569
Treatment at discharge:				
Aspirin (%)	139 (99.3)	71 (100)	68 (98.6)	0.989
Prasugrel (%)	25 (17.7)	11 (15.3)	14 (20.3)	0.577
Ticagrelor (%)	53 (37.6)	33 (45.8)	20 (29.0)	0.059
Clopidogrel (%)	57 (40.4)	28 (38.9)	29 (42.0)	0.891
Dual antiplatelet therapy	113 (80.1)	60 (84.5)	53 (75.7)	0.347
Oral anticoagulation regimen (%)	28 (18.8%)	12 (15.5)	18 (24.3)	0.254
Direct oral anticoagulant (%)	14 (9.4)	4 (5.4)	10 (13.5)	0.118
Vitamin K antagonist (%)	14 (9.4)	6 (8.6)	8 (11.6)	0.756
ACEI or ARB (%)	132 (95.7)	68 (97.1)	64 (94.1)	0.650
Beta‐Blocker (%)	130 (94.2)	66 (94.3)	64 (94.1)	1.000
Statins (%)	132 (95.7)	68 (97.1)	64 (94.1)	0.650
Diuretics (%)	10 (7.2)	6 (8.6)	4 (5.9)	0.779

Abbreviations: ACAD, atherosclerotic coronary artery disease; ACEI, angiotensin‐converting enzyme inhibitor; ARB, angiotensin receptor blockers; DAPT, Dual antiplatelet therapy; DOAC, Direct oral anticoagulation; ECMO, Extracorporeal membrane oxygenation; IABP, Intra‐aortic Balloon Pump; LVEF, Left ventricular ejection fraction; MI, Myocardial Infarction; OAC, Oral anticoagulation; TIMI, Thrombolysis in myocardial infarction; VKA, Vitamin K antagonists.

There were no significant differences in peak troponin or CPK levels between the two groups. Left ventricular ejection fraction at hospital discharge was comparable in both groups (48 ± 10% vs. 49 ± 10%, *p* = 0.569). At discharge, dual antiplatelet therapy was widely prescribed (84.5% vs. 75.7%, *p* = 0.437), with 15.5% of the patients receiving an oral anticoagulation regimen in the CAE with coexisting ACAD group and 24.3% in the isolated CAE group (*p* = 0.254).

### 3.1 Long‐Term Outcomes

**Figure 1:**
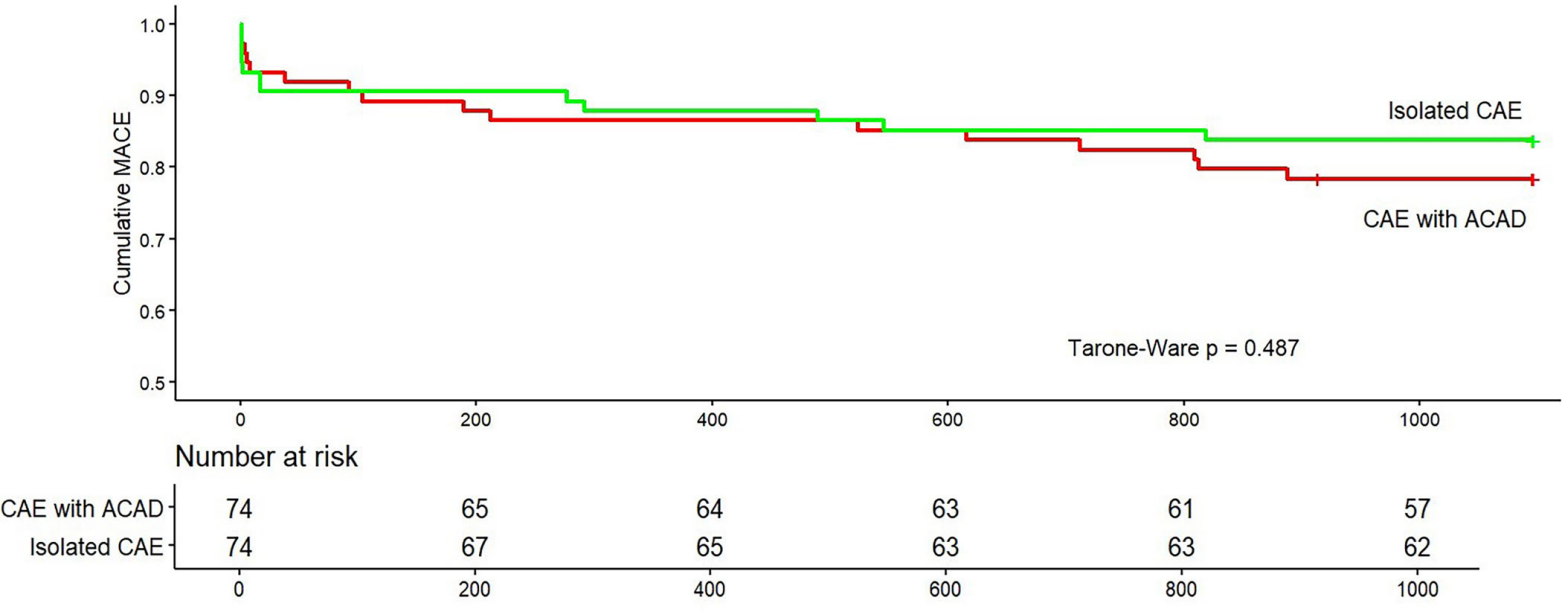
Long‐term MACE‐free survival of patients with STEMI and ectatic infarct related artery according to the presence of atherosclerotic coronary artery disease. [Color figure can be viewed at wileyonlinelibrary.com]

Figure 1 presents the Kaplan–Meier curves for 3‐year cumulative MACE‐free survival stratified by the presence of concomitant ACAD. At the end of follow‐up, the overall MACE‐free survival rate was 85.1%, with no statistically significant difference between the isolated CAE group and the CAE with coexisting ACAD group (83.8% vs. 78.4%, *p* = 0.487). Eight patients (5.4%) experienced recurrent acute coronary syndrome, including three cases of stent thrombosis: four patients in the isolated CAE group and four in the CAE with coexisting ACAD group.

## 4 Discussion

The major findings of this study evaluating a large series of patients with STEMI and ectatic infarct‐related artery disease with CAE are as follows:

1. Atherosclerosis was observed in half of the patients with STEMI with ectatic infarct related artery.
2. Patients with coexisting ACAD were significantly older than those with isolated CAE; however, no significant differences were observed in medical history or cardiovascular risk factors.
3. Coronary angioplasty was more frequently performed in the CAE with coexisting ACAD group with a higher stenting rate, whereas patients with isolated CAE experienced a trend toward more distal embolization.

To the best of our knowledge, this is one of the few mechanistic studies presenting the clinical features, periprocedural management, and prognosis of patients with STEMI related to CAE stratified by the presence or absence of ACAD [7].

The frequent coexistence of CAE with CAD and the histopathological findings resembling those of atherosclerosis have led to the conclusion that the mechanism underlying the pathogenesis of CAE may be a variant of atherosclerosis. However, non‐atherosclerotic types of CAE have been identified where the vessel intima remains intact, and significant degeneration of the media is characterized by the replacement of smooth muscle cells with hyalinized collagen [12, 13]. Several susceptibility genes have been identified, indicating a potential genetic predisposition to coronary ectasia [14]. Coexistence of CAE with other conditions, such as bicuspid aortic valve [15], Kawasaki disease and other vessels aneurysms such as ascending aortic aneurysms or abdominal aortic aneurysms [14, 16], corroborates the conception of isolated CAE as the manifestation of a systemic disease.

Conversely, arteries with CAE and coexisting atherosclerosis exhibit positive remodeling, characterized by the expansion of the area within the external elastic membrane, which may contribute to the development of CAE [17]. These arteries also display significant destruction and reduction of medial elastic fibers, along with disruptions of both the internal and external elastic laminae [18]: angiographically, CAE with coexisting ACAD is often recognized by diffuse but not homogenous dilation.

We showed that 50% of the patients had CAE with ACAD, in agreement with some previously published data [14, 19]. Retrospective studies that systematically analyzed angiograms have shown higher prevalences of atherosclerotic disease [20]; however, these studies do not involve the same population, as our investigation exclusively concerned patients presenting with myocardial infarction.

Angiography only provides a luminal silhouette and may miss early atherosclerotic changes. While those changes might be detectable with advanced imaging modalities such as IVUS [21] or OCT [22], the applicability of these techniques is also limited in the context of coronary ectasia. In vessels with markedly increased diameters, both IVUS and OCT may fail to achieve complete catheter‐wall apposition, while incomplete blood clearance can hinder detailed characterization of the entire vessel wall, potentially resulting in underestimation or misinterpretation of structural abnormalities. In Yu et al, the maximal measured diameter was 8.64 mm, which may limit the applicability of the technique in the largest aneurysms [22].

We found that patients presenting with myocardial infarction and isolated coronary ectasia were younger than those with ACAD. Younger patients in the “non‐atherosclerotic” group may not yet have developed angiographically detectable disease, thus confounding the interpretation of etiological differences. However, we deliberately applied a strict definition for this group, including only patients without any angiographic evidence of atherosclerotic plaques in any coronary segment. The complete absence of atherosclerotic changes in the other vessels further supports the hypothesis that the ectasia observed in this group is not simply a precursor of diffuse atherosclerosis but may represent a different vascular pathology.

Slow flow and distal embolization are feared complications of direct PCI in the context of STEMI. Our study confirmed suboptimal epicardial revascularization in both groups, with TIMI flow grade 3 achieved in just over two‐thirds of patients in both groups, influenced by the large thrombus burden. Procedurally, higher rates of angioplasty and stenting during primary PCI in patients with CAE and coexisting ACAD are consistent with the need to treat plaque ruptures with stenting. Conversely, thromboaspiration was significantly more common in the isolated CAE group, occurring in nearly half of the patients, with GPI administered in 63.5% of cases. These findings highlight differences in the underlying pathophysiology, including probable differences in thrombus burden and composition. Thus, a tailored treatment approach should be employed for each group, anticipating slow flow and distal embolization with deferred stenting and an adapted pharmacological strategy [23].

### 4.1 Long‐Term Outcomes

Because dilatation of the coronary arteries disturbs coronary flow, thereby increasing blood viscosity and activating coagulation, CAE may be a high‐risk lesion that causes acute coronary events [24, 25]. Whether CAE influences the clinical course and prognosis in this high‐risk clinical setting remains debated [4, 24, 25]. The clinical course in our study appeared to be independent of coexisting coronary atherosclerotic disease. At the 3‐year follow‐up, the survival rate ranged from 82.4% in the CAE with coexisting significant difference (*p* = 0.37). This suggests that in patients with isolated CAE, despite undergoing fewer invasive procedures, including fewer angioplasties with stenting during the acute phase, mortality outcomes were similar for up to 3 years.

Furthermore, the enhanced thrombogenicity of CAE suggests the potential benefit of long‐term pharmacological agents for modulating the coagulation cascade to prevent CAE‐related coronary events. This raises the question of antithrombotic strategies for these patients. Although angioplasty with stenting in the STEMI setting requires dual antiplatelet therapy for at least 1 year, the use of long‐term anticoagulants remains controversial. Indeed, a few studies have suggested that patients who experience STEMI with CAE are at a higher risk of recurrent acute coronary syndrome and that anticoagulant therapy could be beneficial for these patients [24, 26]. However, the lack of a clear answer may explain [20, 27] the low anticoagulant prescription rate (approximately 20%, including both VKAs and DOACs), with no significant difference between the two groups. Further studies using specific antithrombotic strategies for each CAE group are required.

### 4.2 Limitations

This study has several limitations. First, this was an observational, retrospective study with an inherent bias. Furthermore, while baseline characteristics and procedural data were well documented, anticoagulation and antiplatelet therapies were initiated at the discretion of each physician, a procedure that is susceptible to selection bias. Eventually, prompt TIMI flow grade 3 restoration and delayed coronary angiography control using systematic intracoronary imaging techniques may have provided interesting data for assessing the mechanism of vessel occlusion. However, this was a real‐world study, and although helpful in some cases, the systematic use of intracoronary imaging for PCI guidance in large CAE settings is often inappropriate.

## 5 Conclusion

Primary PCI of ectatic infarct related artery represents a significant therapeutic challenge owing to the initially high thrombus burden and the frequent occurrence of suboptimal angiographic results. The coexistence of CAE and ACAD implies distinct periprocedural characteristics and further prospective studies are needed to determine optimal therapeutic management at discharge.

## Conflicts of Interest

The authors declare no conflicts of interest.
